# Evolving insights on the role of microglia in neuroinflammation, plasticity, and regeneration of the injured spinal cord

**DOI:** 10.3389/fimmu.2025.1621789

**Published:** 2025-08-19

**Authors:** Emily A. Swarts, Faith H. Brennan

**Affiliations:** ^1^ Department of Biomedical and Molecular Sciences, Queen’s University, Kingston, ON, Canada; ^2^ Centre for Neuroscience Studies, Queen’s University, Kingston, ON, Canada

**Keywords:** microglia, astrogliosis, neurotrauma, demyelination, axon regeneration

## Abstract

Microglia have emerged as central players in the pathophysiology of traumatic spinal cord injury (SCI). The purpose of this brief review is to highlight the evolution of knowledge on the role of microglia in SCI. We explore the initial discovery of macrophages and their role in SCI lesions, followed by how microglia were examined and distinguished from monocyte-derived macrophages. We then discuss findings from studies that mapped and manipulated microglia in experimental SCI, made possible through technological advances in genetic, pharmacological, and bioinformatic approaches. We also highlight the importance of considering how the timing and location of microglia activation shapes neuroinflammation, synaptic plasticity and intraspinal circuit remodelling. Finally, as microglia research continues to flourish, we consider how microglia could be harnessed therapeutically to promote repair and functional recovery of motor, sensory, and autonomic systems after SCI.

## Early descriptions of macrophages in SCI lesions

**Figure 1:**
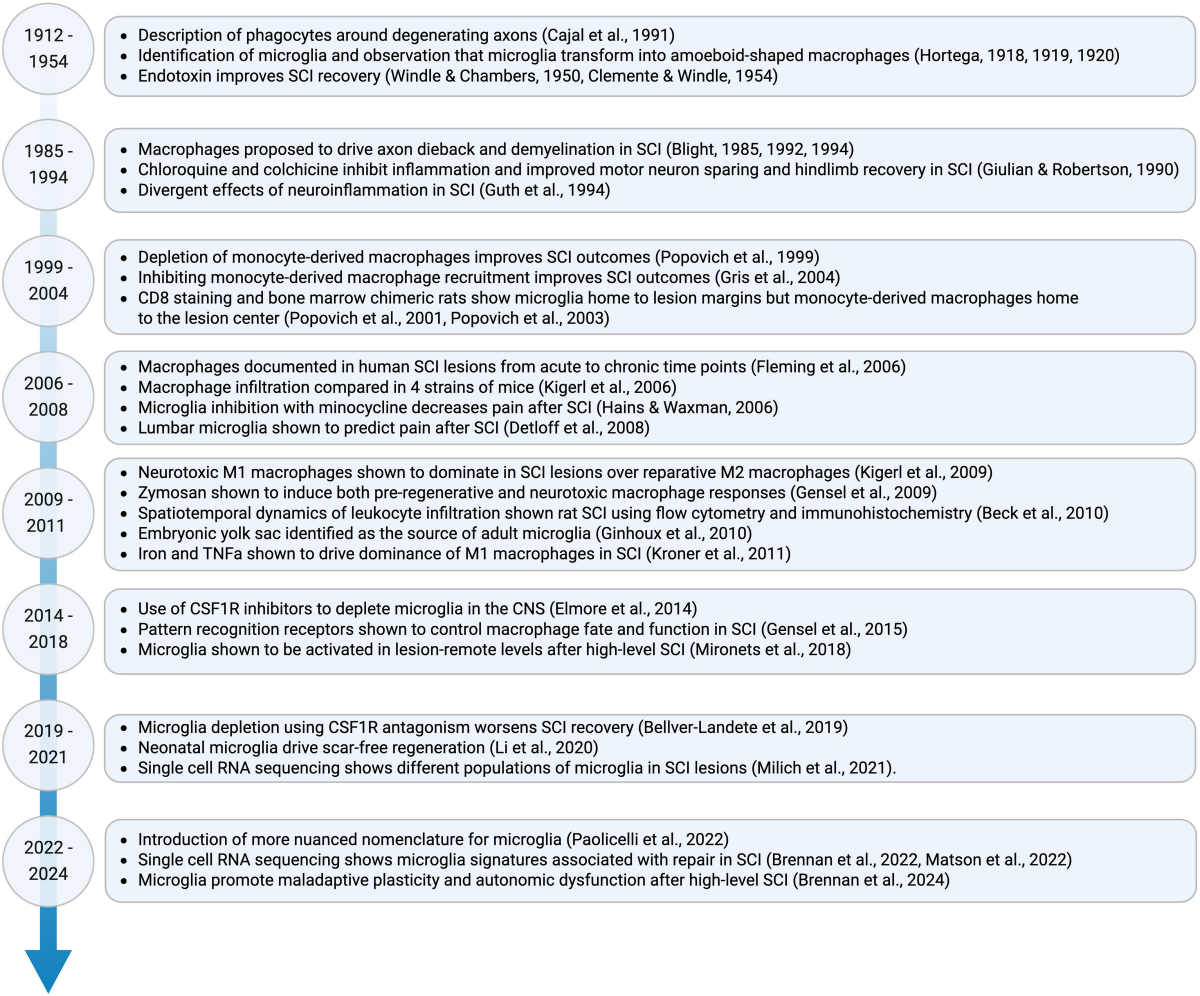
Timeline of major discoveries on the role of microglia in SCI. Due to space restrictions only select papers are shown.

Macrophages are the most abundant immune cell type found in clinical and experimental spinal cord injury (SCI) lesions (1–3). This rich population is derived from at least two phenotypically similar but ontogenetically distinct sources: circulating monocyte-derived macrophages that originate from the spleen and bone marrow, and tissue-resident microglia that originate from the embryonic yolk sac (4–8). Because the macrophage response to SCI is prolific and conserved across species, macrophage-targeting therapies hold great potential to repair the injured spinal cord if the role of both blood-borne and tissue-resident macrophage populations can be deciphered. Research over the last century has made great strides toward this goal (**Figure 1**).

Initial descriptions of macrophages in SCI were made in the early 1900’s by Spanish neuroanatomist Santiago Ramón y Cajal. In spinal tissue sections from cats, dogs, and rabbits with contusion, laceration, or transection SCI, Cajal observed rapid ‘traumatic degeneration’ – dystrophic axon bulbs that were thought to underlie central nervous system (CNS) regeneration failure (9). Cajal remarked that the centers and peripheral stumps of these degenerated and blebbing nerves were a ‘pasture-ground for phagocytes’ (9–12). Cajal’s silver staining techniques were not able to determine the origin, phenotype, or functional repertoire of lesion-associated phagocytes, though he accurately predicted that most of the cells directly around dystrophic axons originated from the blood (12). After over 100 iterations of protocol development, Cajal’s contemporary, Pío del Río Hortega, integrated lithium carbonate with silver nitrate staining and formalin-ammonium bromide fixation methods to precipitate silver carbonate (13). Using this method, the cytoplasmic expansions of cells with a tiny soma and branched processes could be distinguished from astrocytes and neurons in the intact CNS (14, 15). As these cells were smaller than other glia and exhibited shorter, finer processes, they were called microglia (14, 15). Hortega noted that microglia could migrate, phagocytose, and undergo morphological transformation, increasing their soma to become amoeboid-shaped macrophages (16, 17). However, at the time it was impossible to distinguish microglia from infiltrating, monocyte-derived macrophages in CNS lesions, or to determine their functional role in CNS injury. Despite the discovery that CNS lesions were rich in macrophages, neuroimmune research stagnated for the next three decades. This was because the study of glia and phagocytosis was limited to morphological characterizations with insufficient tools to assess function. Also, electrical properties could not be detected in glial or immune cells at the time, making them less attractive to study than neuronal action potentials. Third, glia were still largely considered as ‘connective tissue’ that simply held nervous elements together (18). Fortunately, this view would dramatically change in future years.

## Functional roles for macrophages in SCI repair

In the 1950s, an unexpected discovery highlighted functional interactions between neuronal, immune, and glial cells that rejuvenated neuroimmune research. Injection of Priomen, a crude pyrogen used to study mechanisms of thermal regulation, improved functional recovery after SCI in dogs (19). Macrophage profiles were detected adjacent to newly sprouting nerve fibers, extending their processes around demyelinated axons, with their cell bodies laden with lipid debris months and years post-SCI (19, 20). Studies three decades later in rats with SCI found that injection of bacterial endotoxin also enhanced macrophage accumulation and functional recovery (21). The beneficial effects of macrophages were thought to be mediated by the removal of cellular debris required to stimulate tissue revascularization and reconstruction (21, 22). However, the beneficial effects of endotoxin were augmented by simultaneous injection with anti-inflammatory steroids (21, 22). This was among the first observations showing the divergent effects of neuroinflammatory cells in SCI. Data from subsequent studies in the early 1990’s in different species also showed that the inflammatory response, which was known to involve macrophages, could be harmful to SCI motor, sensory and autonomic recovery (23, 24). For example, chloroquine and colchicine decreased the number of macrophages and improved motor neuron sparing, hindlimb recovery, and bladder function when given to rabbits six hours after ischemic SCI (24), although effects on specific motor or autonomic neuron subtypes were not identified. Similarly, injection of silica dust to suppress macrophage function improved sparing of myelinated axons in the dorsal horn of guinea pigs with lateral compression SCI (23). In the 1980’s and early 1990’s, electron and light microscopy studies of axons in contusion lesions revealed that the number of intact axons decreases over 2–7 days (d) post-injury, coinciding with invasion of macrophages (25, 26). However, the specific macrophage subsets, neurons they interacted with, and intracellular signaling pathways affected by these broad-acting immune-modulatory strategies was not fully understood.

In the 2000’s it became appreciated that intraspinal macrophages have the potential to promote both tissue injury and repair in SCI, and that these seemingly divergent effects are not necessarily mutually exclusive (27–30). The injured spinal cord is rich in damage-associated molecular patterns (DAMPs), including heat shock proteins, necrotic cell debris, extracellular matrix products (fibronectin, hyaluronic acid), high-mobility group box 1, and mRNA, that can activate macrophage pattern recognition receptors (PRRs). Stochastic interactions between DAMPs and macrophage PRRs have the capacity to control the functional fate of monocyte-derived macrophages and microglia in SCI lesions (31, 32). Indeed, the phenotype of intraspinal macrophages changes as the lesion environment evolves (33).

Although more dimensional descriptions of macrophages are now used to better capture the phenotypic and functional heterogeneity of macrophages (34), a linear scale was initially used to describe intraspinal macrophages. Macrophages were often described as being activated on a continuum from ‘pro-inflammatory/M1’ to ‘anti-inflammatory/M2’ macrophages (33). M1 macrophages express more iNOS, CD86 and CD16/32, and are activated by endotoxin, interferon (IFN)-γ and tumor necrosis factor (TNF)-α. M2 macrophages express more CD206, Arginase-1 and CD16, and are activated by IL-4 and IL-13. In SCI, M1 macrophages drive neuron death and axon dieback, whereas M2 macrophages can promote neuron survival and axon outgrowth even across grown-inhibitory gradients containing chondroitin sulphate proteoglycans (33). In line with this, blocking M2 macrophage recruitment worsens motor recovery and increases lesion size (35). The typical ratio of M1:M2 macrophages in SCI is ~50:50 until 7 d post-injury, but unfortunately, M1 macrophages dominate after 14 d post-injury (33, 36), and transplanted M2-polarized macrophages differentiate into M1 macrophages (12, 33). The reason that harmful M1 macrophages ultimately dominate SCI lesions was a mystery until a seminal study showed that intraspinal iron and TNF are powerful signals that prevent phagocytosis-mediated conversion from M1 to M2 macrophages (37).

However, pro-inflammatory macrophage activation is not exclusively detrimental. This was demonstrated by combining intraspinally injected zymosan, a glucan polysaccharide found in yeast and potent macrophage activator, with transplantation of dorsal root ganglion (DRG) cells into the same spinal cord (38). Zymosan triggers a florid macrophage response and drives DRG axon outgrowth through the release of macrophage-derived neurotrophins and growth factors [e.g., brain-derived neurotrophic factor (BDNF), ciliary neurotrophic factor (CNTF), and glial cell line-derived neurotrophic factor (GDNF)] (29, 38, 39). However, enhanced axonal outgrowth induced by zymosan occurs concurrently with axon loss and neuron death near reactive macrophages (38). This is likely because zymosan can have paradoxical roles depending on which PRR(s) it activates. Specifically, zymosan can bind to both dectin-1, a C-type lectin receptor (CLR), and toll-like receptor 2 (TLR2). The activation of dectin-1 on intraspinal macrophages drives zymosan-induced axonal dieback and increases lesion size (40). Conversely, the activation of TLR2 using a TLR2 antagonist, which also triggers macrophage activation, increases axon density and reduces axon retraction from the lesion site (40, 41). These data are reminiscent of observations made decades earlier using crude pyrogens and endotoxin (19–22), which activate TLR2. The potential to manipulate macrophage functional plasticity to promote repair of the injured spinal cord is the subject of several excellent reviews (12, 42–49), although monocyte-derived macrophages and microglia are often considered together.

## Mapping the location of monocyte-derived macrophages vs. microglia in SCI

**Figure 2:**
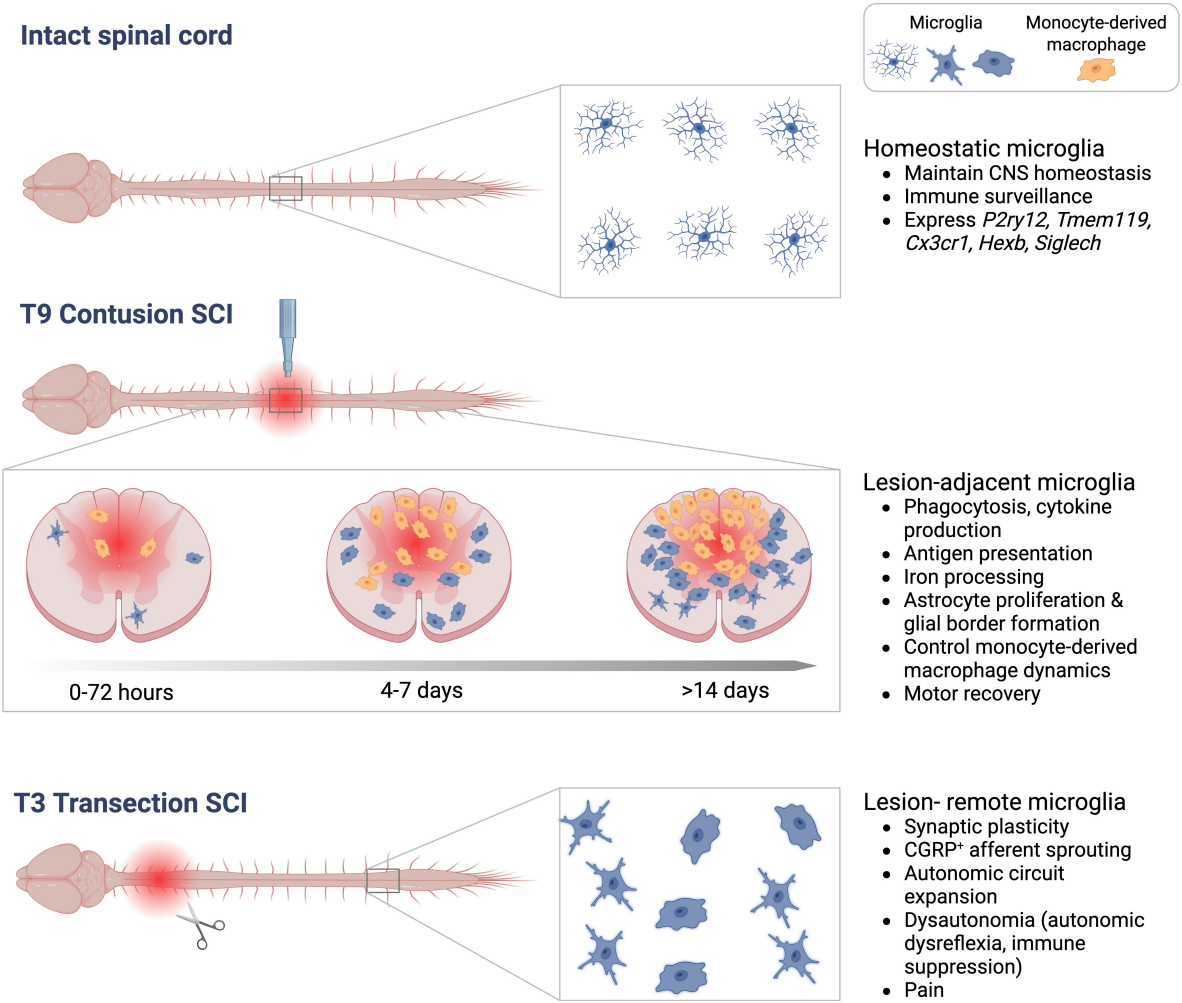
Schematic showing different activation states and functions of microglia. Top<: Microglia tile throughout the intact spinal cord and exhibit a ramified morphology. Middle: Microglia adjacent to a T9 contusion SCI become phagocytic and stimulate cytokine production, coordinate astrogliosis and the inflammatory response to drive motor recovery. Microglia and monocyte-derived macrophages home to specific regions of SCI lesions in a time-dependent manner. Bottom: After a T3 transection SCI, microglia in lesion-remote thoracic and lumbar segments drive maladaptive plasticity after high-level SCI.

As it became evident that macrophages had significant but complex roles in SCI pathophysiology, subsequent efforts sought to better understand macrophage heterogeneity, beginning with distinguishing microglia from monocyte-derived macropahges. Adoption of specific tools, including targeted antibody labeling, bone marrow chimeras, and transgenic reporter mice, enabled more precise mapping of the niches that monocyte-derived macrophages vs. microglia occupy within SCI lesions (**Figure 2**). Monoclonal antibody staining to CD8 showed that hematogenous macrophages home to central necrotic regions of lesion cavitation after rat spinal cord injury (50). Bone marrow chimeric rats demonstrated that microglia are activated rapidly after SCI and are present around the injury site, whereas monocyte-derived macrophages exclusively infiltrate the central gray matter lesion, and to a lesser extent the subpial white matter, peaking recruitment around 7 d post-SCI (51). Lys-EGFP-ki mice (which express enhanced green fluorescent protein (EGF) in mature myeloid lineage cells but not microglia) showed that at six weeks after compression SCI, monocyte-derived macrophages reside in the lesion epicentre, but microglia are at the lesion margins (52). Lys-EGFP-ki mice were also used to show that microglia are the first macrophage population to contact degenerating axons *in vivo* (within minutes). After ~ 3 d post-injury, monocyte-derived macrophages become the main cell type contacting dying axons, but they process phagocytic material less effectively than microglia (53). Studies using Cx_3_cr1^gfp/+^>WT bone marrow chimeric mice also confirmed that monocyte recruitment is delayed relative to microglia, peaking around 7d post-SCI, and that these cells home to the central gray matter (5, 54). More recent studies using tamoxifen-inducible conditional transgenic reporter mice (Cx_3_cr1^creER::R26-TdT^) to selectively label microglia showed that microglia rapidly die but then proliferate extensively during the first two weeks post-SCI (55). These proliferating microglia home to the interface between infiltrating leukocytes and astrocytes (55). The homing of monocyte-derived macrophages and microglia to distinct alcoves of SCI lesions suggests that the developmental origin of macrophages dictates which lesion-associated ligands they are exposed to, and their functional effects on surrounding tissue (51).

## Distinguishing the function of monocyte-derived macrophages and microglia using targeted cell depletion strategies

Although both monocyte-derived macrophages and microglia have the capacity to drive repair or secondary injury, the use of more precise strategies to deplete specific macrophage populations provided evidence that blood-borne macrophages are mostly harmful to the injured spinal cord, whereas tissue resident microglia are mostly beneficial. Intravenously injected liposome-encapsulated clodronate depletes monocyte-derived macrophages and improves hindlimb locomotion, preserves myelinated axons, decreases cavitation, and enhances axon sprouting in the lesion (56–58). The tissue damage and macrophage activation induced by zymosan can also be partially reversed by injecting clodronate-encapsulated liposomes (38). Radiation bone marrow chimeric rats also confirmed that hematogenous macrophages are the principal effectors of zymosan-induced axonal pathology (59). *In vivo* studies and time-lapse imaging in cultured dorsal root ganglion neurons showed that monocyte-derived macrophages physically interact with dystrophic axons and drive their retraction (60). Like hematogenous macrophage depletion, blocking recruitment of circulating myeloid cells into SCI lesions via intravenous injection of a neutralizing antibody to CD11d integrin or CD49d/CD29 integrin improves motor performance, myelin preservation, and axon sparing in rodent SCI (61–63).

In 2014, colony stimulating factor 1 receptor (CSF1R) inhibitors became available to deplete microglia without depleting monocyte-derived macrophages (64). These tools have now been used by several groups to interrogate the role of microglia in contusion SCI. The data show that pharmacological microglia depletion impairs motor recovery by disrupting several naturally occurring neuroprotective processes (55, 65, 66). Microglia-dependent protective functions include: JAK/STAT3-dependent astroglial proliferation and protective astroglial border formation, promoting neuronal survival, releasing neurotrophins, axon regeneration, and oligodendrocyte precursor cell survival (55, 65–69) (**Figure 2**). Microglia depletion also significantly delays the entry of monocyte-derived macrophages into spinal lesions. When monocyte-derived macrophages do arrive, they disperse throughout ventrolateral white matter regions that would normally be spared, and hinder motor recovery (66). This is in line with data showing that blocking the centripedal migration and sequestration of monocyte-derived macrophages to the central lesion core by worsens tissue sparing and functional recovery from SCI (70–72). Increasing microglial proliferation by local delivery of macrophage-colony stimulating factor (M-CSF) reduces lesion size and enhances functional recovery (55). Similarly, engineering microglia to overexpress BDNF, using Cx_3_cr1^creER::^BDNF or Tmem119::BDNF transgenic mice, reduces inflammation, neuronal death, and increases angiogenesis and motor recovery in mice with T10 crush SCI (67). A protective role of microglia on spinal vasculature was also demonstrated in an aortic cross-clamp model of ischemic SCI. Serial injections of lipopolysaccharide (LPS) prior to SCI ‘prime’ microglia and prevents ischemia-induced paralysis; LPS-induced neuroprotection is reversed by microglia depletion (73). IL-1-dependent microglia-endothelial cell interactions are critical in mediating this neuroprotective program (73). Collectively, studies using microglia-specific depletion strategies consistently show that, in contrast to monocyte-derived macrophages, microglia drive repair and regenerative processes after SCI.

### Transcriptional responses of microglia to SCI

Since boosting the beneficial functions of long-lived microglia *in vivo* could be a novel therapeutic strategy for SCI, it is critical to understand the mechanisms through which microglia drive CNS repair. Research in recent years has taken advantage of RNA sequencing technologies to provide more granular insight as to how microglia coordinate inflammation, neuroprotection, and tissue repair in SCI. Bulk RNA sequencing of spinal cord homogenates showed that >50% of the top 1000 genes that are increased by SCI require microglia presence (66). Gene ontology analysis showed that these genes are responsible for microglia proliferation, phagocytosis, cytokine production, endocytosis, and/or protein secretion (e.g., *Aif1, Ccl2, Ccl3, Ccl5, CD14, Cd36, Osm, Pycard, Syk, Tgfb1, Tlr2, Tlr4, Tnf, Trem2*) (66, 74). The beneficial effects of microglia in SCI are partly mediated through phagocytosis and cytokine production, since the worsened phenotype of microglia-depleted mice can be rescued by reconstituting the lesion environment with recombinant CCL2 and a TLR2 agonist, effectively reprograming monocyte-derived macrophages to become less destructive (66). These data are in line with observations that efficient phagocytic clearance of myelin debris and apoptotic cell material is required for tissue repair, remyelination, and axon regeneration after SCI (44–47).

Single cell RNA sequencing datasets also show that microglia coordinate SCI repair by dynamically changing their transcriptional phenotype. In the intact spinal cord, microglia mainly express homeostatic genes, including *P2ry12, Tmem119, Hexb, Siglech*, and *Cx3cr1* (66, 75, 76). However, microglia in the injured spinal cord adopt several injury-associated transcriptional phenotypes, including genes that control cell lipid phagocytosis (e.g., *Cd68, Clec7a, Ctsd, Ctsz, Trem2, Apoe*), iron processing (*e.g., Fth1, Ftl1*), interferon production (e.*g., Ifit1, Ifit2, Ifit 3, Irf7*), and antigen-binding and processing (*e.g., H2-Ab1, H2-Eb1, CD74, Cd93, Cd38*) (66, 75–78). These phenotypes shift in proportion over time, but can be found in acute (1–3 d), subacute (7 d) and chronic (one month) time points (66, 75). Evaluating the transcriptional profile of other cell types in the lesion shows that microglia are also required for astrocytes to increase genes that drive cytoplasmic translation, response to interleukin-4, and immune responses (e.*g. Tmsb4x, Fth1, Apoe*) (66). Transcriptional analysis of monocyte-derived macrophages shows that without microglia present, monocyte-derived macrophages express more genes that could promote inflammation and neurotoxicity (e.g., *Cd86, Cd36, Clec12a)* (33, 66, 79). The induction of these transcriptional programs by microglia explains why astroglial and monocyte-derived macrophage responses to SCI are disrupted without microglia.

CSF1R inhibition combined with single cell RNA sequencing also revealed how microglia control axon regeneration in the injured young vs. adult CNS. Mice at postnatal day two exhibit scar-free healing and axon regeneration across the lesion site (69). Microglia are critical for neonatal spinal cord regeneration, as microglia depletion prevents axon regeneration across the lesion site (69). Single cell RNA sequencing showed that neonatal microglia secrete extracellular matrix bridge proteins (e.g. *Fn1, Thbs1*) that ligate the crushed spinal cord ends, then produce peptidase and endopeptidase inhibitors (e.g. *Cstb, Stfa1, Serpin6a, Anxa1*) that drive resolution of inflammation (69). Transplantation of neonatal microglia or peptidase inhibitor-treated microglia into adult lesions improves axon growth and tissue repair (69). Regeneration-associated bridging microglia are much less abundant in the adult spinal cord and express higher levels of CD68 and lower levels of P2y12, which is thought to dampen their ability to promote regeneration in the adult spinal cord (69, 80).

Interestingly, a recent study showed that if microglia are depleted and then allowed to repopulate the inflammatory environment of chronic SCI lesions, they return with a more pro-inflammatory and pro-regenerative phenotype than the original microglia (81). In this study, CSF1R was inhibited from 7–9 weeks post-SCI and then the inhibitor was withdrawn from week 9–12 to allow microglia to repopulate (81). Microglia depletion reduced expression of inflammatory genes (e.g. *C1qb, Ccl12*) (81). In comparison, forcing microglia turnover increased extracellular matrix genes (e.g. *Ncam1, Cadm3, L1cam*) and neuronal transcripts (e.g. *App, Nptn, Nf1, Nrxn1*), which were associated with increased density of β3-tubulin^+^ axons in the lesions (81).

We anticipate that ongoing sequencing studies will continue to shed light on mechanisms of biological heterogeneity as a function of time post-injury, injury level, injury severity, proximity to the lesion, biological sex, age, and other environmental or therapeutic factors. These data could then be harnessed to provide new microglia-dependent targets that could be co-opted to develop tailored microglia-dependent therapeutics.

## Lesion-remote microglia shape intraspinal plasticity after SCI

Although most research has focused on lesion-adjacent microglia and their role in neuroinflammation, microglia distant to the lesion can also become activated and shape spinal circuitry to affect functional outcomes from SCI (**Figure 2**). The role of microglia in synaptic plasticity and circuit remodeling was recently shown to be critical for the development of autonomic dysregulation after SCI (82). A high-level SCI above the major sympathetic outflow (spinal level T6) disinhibits sympathetic preganglionic neurons (SPNs) from descending brainstem control. Consequently, remarkable synaptic plasticity, axonal sprouting and autonomic circuit expansion occurs within circuits that control lymphoid and endocrine organs (83–84). This leads to a condition called dysautonomia, which manifests in the cardiovascular system as autonomic dysreflexia, in the immune system as immune-depression syndrome, and in the endocrine system as metabolic syndrome (83, 84). In T3 transection SCI, microglia increase in number and adopt hypertrophic, amoeboid-shaped morphologies in thoracic and lumbar spinal segments centimeters away from the lesion (3, 82, 85, 86). Microglia activation in lesion-remote regions is triggered by the activity of disinhibited glutamatergic interneurons; silencing excitatory neuron activity by blocking Vglut2 activity, or blocking calcium channel α2δ-1 signaling, prevents microglia hyperplasia and hypertrophy (82). These interventions also prevent maladaptive synaptic plasticity, circuit formation and dysautonomia (82, 87, 88).

To determine if microglia have a causal role in maladaptive plasticity and dysautonomia, microglia were depleted pharmacologically using CSF1R antagonism or genetically using Cx_3_cr1^creER^xR26^iDTR^ mice. These experiments showed that microglia depletion blocks structural and functional plasticity of autonomic circuits after high-level SCI (82). Specifically, microglia depletion prevents SCI-induced excitatory synaptogenesis and loss of inhibitory synapses, decreases sprouting of lumbar CGRP^+^ afferents, and prevents the expansion of neuronal circuits that innervate lymphoid and endocrine tissues (82). Consequently, indices of dysautonomia (i.e., autonomic dysreflexia, splenic atrophy, antigen-specific antibody production), are also improved by microglia depletion in high-level SCI. Mechanistically, microglia strip inhibitory synapses from SPNs and the interneurons they connect to, lowering their threshold for activation and excitatory circuit formation. The Trem2 receptor is at least partially required for this response (82). Other studies have shown that inhibition of soluble TNFα, which is predominantly produced by microglia, prevents maladaptive structural plasticity and autonomic dysregulation after high-level SCI (85, 89).

Lesion-remote microglia are also thought to drive thermal and mechanical hypersensitivity post-SCI. Activation of lumbar microglia is associated with phosphorylation of p38 MAP kinase, elevated TNFα and IL-1β levels, and induction of allodynia after SCI (90). The inhibition of lesion-remote microglia using minocycline prevents hyperresponsiveness of lumbar dorsal horn neurons, p38 MAP kinase and blocks SCI-induced pain (91). Thus, in designing strategies to manipulate microglia therapeutically, it is important to not only consider the protective role of lesion-adjacent microglia in coordinating neuroinflammation, but also the pathological role of lesion-remote microglia in aberrant signaling that drives dysautonomia and pain.

## The future: microglia-targeting strategies to repair the injured spinal cord

There are now several genetic and pharmacological approaches being actively explored to manipulate microglia to promote tissue repair and functional recovery from SCI. Microglia transplantation (69, 92, 93) in specific CNS regions is possible through local intraparenchymal injections, although whether their phenotype and function remains long-term is unknown. A more targeted approach is to use lipid-polymer-hybridized-nanoparticles (LPNPs) to deliver siRNA within defined CNS regions to modify microglial gene expression (94). This technique harnesses the fact that microglia are the primary phagocytes in the CNS, and selectively phagocytose biocompatible nanoparticles loaded with siRNA and either Rhodamine B or Alexa555-conjugated gold nanoparticles tracers, allowing microglial fate-mapping alongside gene manipulation (94). However, this technique requires fully functional phagocytosis pathways (i.e., ‘find me’, and ‘eat me’ signals), which may themselves be modified by pathology.

An alternative approach is to use adeno-associated viral (AAV) vectors containing, for example, Iba1 promoter regions to transduce microglia *in vivo* (95–97). AAV viral vectors have successfully modified microglial gene expression and disease outcomes in various neurodegenerative diseases and peripheral neuropathies (95–100). Since SCI has a less complex progression staging and timing of diagnosis than these conditions, it should be possible to time the delivery of AAV therapies to target specific microglia-dependent neuroinflammatory events. However, since SCI lesions have a larger contingent of peripheral immune cells than chronic neurodegenerative lesions, AAV technologies may not be as effective in distinguishing and targeting microglia vs. monocyte-derived macrophages in SCI. However, a recent study used a combinatorial genetic and surgical strategy to chronically target microglia with region specificity, without affecting peripheral macrophages (101). Specifically, a tamoxifen metabolite (endoxifen) was administered to Cx_3_cr1^creERT2^ or TMEM119^creERT2^ mice. Sustained microglia gene manipulation was achieved by delivering endoxifen through osmotic pumps attached to fine cannulas made of stainless steel or microfluidic polymer fibers (101).

Microglia are also central components of various other therapeutic strategies in development for SCI. For example, the gut microbiome influences microglial immunosurveillance, phenotype, and synaptic remodeling, suggesting that microglia could also be co-opted non-invasively through strategies targeting the gut-brain axis (102, 103). Epigenetic changes (e.g. DNA methylation, histone deactylation) impact microglia responses and represent a novel therapeutic avenue (104). Microglia-targeting therapies have also been shown to boost the efficacy of other interventions, such as rehabilitation training (105). We expect that in future years, these and many other strategies centered on microglia biology will emerge as flourishing fields to enhance recovery after SCI, and potentially other types of CNS trauma.
